# Elaeocarpusin Inhibits Mast Cell-Mediated Allergic Inflammation

**DOI:** 10.3389/fphar.2018.00591

**Published:** 2018-06-07

**Authors:** Min-Jong Kim, Yeon-Yong Kim, Young-Ae Choi, Moon-Chang Baek, Byungheon Lee, Pil-Hoon Park, Tae-Yong Shin, Taeg Kyu Kwon, Dongwoo Khang, Sang-Hyun Kim

**Affiliations:** ^1^CMRI, Department of Pharmacology, School of Medicine, Kyungpook National University, Daegu, South Korea; ^2^Department of Molecular Medicine, School of Medicine, Kyungpook National University, Daegu, South Korea; ^3^Department of Biochemistry and Cell Biology, School of Medicine, Kyungpook National University, Daegu, South Korea; ^4^College of Pharmacy, Yeungnam University, Gyeongsan, South Korea; ^5^College of Pharmacy, Woosuk University, Jeonju, South Korea; ^6^Department of Immunology, School of Medicine, Keimyung University, Daegu, South Korea; ^7^Department of Physiology, School of Medicine, Gachon University, Seongnam, South Korea

**Keywords:** allergic inflammation, anaphylaxis, elaeocarpusin, histamine, mast cells

## Abstract

Mast cells are major effector cells for allergic responses that act by releasing inflammatory mediators, such as histamine and pro-inflammatory cytokines. Accordingly, different strategies have been pursued to develop anti-allergic and anti-inflammatory candidates by regulating the function of mast cells. The purpose of this study was to determine the effectiveness of elaeocarpusin (EL) on mast cell-mediated allergic inflammation. We isolated EL from *Elaeocarpus sylvestris* L. (Elaeocarpaceae), which is known to possess anti-inflammatory properties. For this study, various sources of mast cells and mouse anaphylaxis models were used. EL suppressed the induction of markers for mast cell degranulation, such as histamine and β-hexosaminidase, by reducing intracellular calcium levels. Expression of pro-inflammatory cytokines, such as tumor necrosis factor-α and IL-4, was significantly decreased in activated mast cells by EL. This inhibitory effect was related to inhibition of the phosphorylation of Fyn, Lyn, Syk, and Akt, and the nuclear translocation of nuclear factor-κB. To confirm the effect of EL *in vivo*, immunoglobulin E-mediated passive cutaneous anaphylaxis (PCA) and ovalbumin-induced active systemic anaphylaxis (ASA) models were induced. EL reduced the PCA reaction in a dose dependent manner. In addition, EL attenuated ASA reactions such as hypothemia, histamine release, and IgE production. Our results suggest that EL is a potential therapeutic candidate for allergic inflammatory diseases that acts via the inhibition of mast cell degranulation and expression of proinflammatory cytokines.

## Introduction

The worldwide prevalence and severity of allergic diseases, including atopic dermatitis, allergic rhinitis, and asthma, have increased dramatically over the past decade, especially in developed countries. Allergy is one of the most common chronic diseases, which lasts a long time and occurs frequently (Larsen et al., 2016). Mast cells play a prime role in inducing early- and late-phase IgE-mediated allergic inflammation. They express the high-affinity IgE receptor (Fc𝜀RI) that reacts with specific IgE molecules and can secrete a wide range of biological mediators, such as pre-formed granule-related mediators (histamine and proteases), lipid-derived mediators (leukotriene C_4_ and prostaglandin D_2_), and *de novo* synthesized pro-inflammatory cytokines, chemokines, and growth factors (Galli et al., 2008). Among these mediators, mast cell-derived histamine affects various biological processes, such as inflammation of the surrounding tissues, vasodilation, mucous secretion, and bronchoconstriction (da Silva et al., 2014). Thus, mast cells are a critical target for the treatment of allergic inflammation.

The signaling pathways regarding mast cell degranulation has been largely studied (Alsaleh et al., 2016). Phosphorylation of Src family kinases (Lyn, Syk, and Fyn) is induced cross-linking of Fc𝜀RI (Huber, 2013). Phosphorylation of Lyn and phospholipase C (PLC)γ induces calcium mobilization after inducing mast cell degranulation through granulosa cell fusion (Holowka and Baird, 2015; Chelombitko et al., 2016). As mentioned above, mast cells influence the late reactions of allergic inflammation by releasing pro-inflammatory mediators such as TNF-α and IL-4. TNF-α is a potent inflammatory mediator that is the central of inflammation mediated by the innate immune system, inclusive of the initiation of cytokine production, activation or expression of adhesion molecules, and promotion of growth (Turner et al., 2014). IL-4 also plays a significant task in chronic allergic inflammation (Cheng and Locksley, 2014). Thus, the inhibition of TNF-α and IL-4 is considered to be the most important therapeutic step in allergic inflammation. These cytokines are largely regulated by NF-κB (Hoesel and Schmid, 2013).

Tannins can be divided into four main groups according to their structural properties: gallotannins, ellagitannins, condensed tannins, and complex tannins. They are secondary metabolites that are water-soluble phenolic with molar masses of 300 to 3000 Da (Erdelyi et al., 2005; Li et al., 2006). A number of oriental medicinal plants are rich in tannins, which are responsible for their medicinal usages. Various tannins have been isolated from these medicinal plants, and the chemical structures have been determined (Ropiak et al., 2017). Ellagitannins have been reported to show various biological effects from anti-inflammatory to anti-bacterial effects (Kim H.H. et al., 2014; Kiss and Piwowarski, 2016; Shimozu et al., 2017). Elaeocarpusin (EL) is an ellagitannin, in which one of the aromatic rings is modified via oxidation to a dehydrohexahydroxydiphenoyl ester group(s). Previously we isolated EL from *Elaeocarpus sylvestris* L. (Elaeocarpaceae) (Takashi et al., 1986; Lee et al., 1990). *Elaeocarpus* comprises approximately 350 species that are distributed worldwide (Brambach et al., 2016). Many species of *Elaeocarpus* have been shown to exhibit beneficial pharmacological activities. Especially, various extracts (petroleum ether, benzene, chloroform, acetone, and ethanol) of *E. sphaericus* fruits demonstrated the efficacy of *E. sphaericus* against bronchial asthma through mast cell stabilizing activity (Singh et al., 2000). *E. sylvestris* is an evergreen tree species distributed in tropical and subtropical regions of Jeju Island in South Korea, Southern China; Okinawa and Kyushu, Japan; and Taiwan (Bae et al., 2017). It is a tree with fast growing, evergreen, broad leafy wood, strong adaptability, easy to breed, and good water conservation capacity (Li et al., 2016). To our knowledge, this is the first evidence for the effects of EL isolated from *E. sylvestris* on mast cell-mediated allergic inflammation.

## Materials and Methods

For a complete description of the methods used in the *in vitro* and *in vivo* experiments, please see the Supplementary Materials.

### Isolation of EL

**FIGURE 1:**
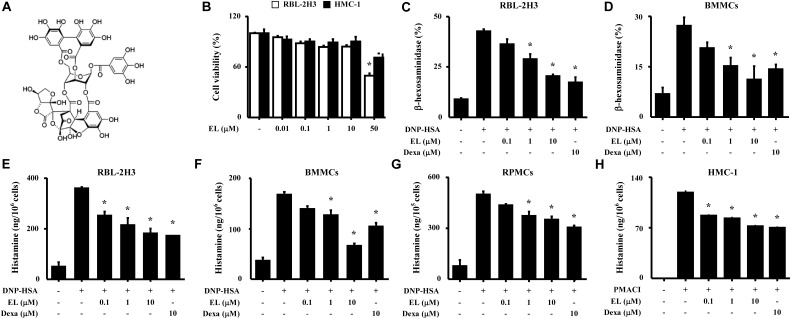
Effects of EL on the degranulation of mast cells. **(A)** Chemical structure of EL. **(B)** RBL-2H3 cells (6 × 10^4^/well) were pretreated with or without EL for 12 h and then incubated with 1 mg/mL MTT for 2 h. The absorbance intensity was measured using a spectrophotometer. **(C–G)** RBL-2H3 cells, BMMCs (5 × 10^5^/well), and RPMCs (2 × 10^4^/well) were sensitized with anti-DNP IgE (50 ng/mL). After overnight incubation, the cells were pretreated with or without drugs, including EL and Dexa, for 1 h or 30 min and then challenged with DNP-HSA (100 ng/mL). **(H)** HMC-1 cells (1 × 10^6^/well) were pre-incubated with EL for 30 min prior to incubation with PMACI for 30 min. β-Hexosaminidase and histamine levels were detected using a spectrophotometer or a fluorescent plate reader, respectively. Graph data represent the mean ± SEM of three independent experiments. ^∗^*p* < 0.05. Dexa, dexamethasone.

Leaves of *E. sylvestris* were purchased from Yak-Ryung-Si Market in Daegu, South Korea and a sample voucher (YU00197) confirmed by Professor Seung-Ho Lee of Yeungnam University was deposited with the Natural Product Research Institute of Pharmacy College. Leaves (13 kg) were extracted with acetone–water [9:1 (v/v)] at room temperature. The extract was concentrated under reduced pressure to yield a thick green precipitate consisting mainly of chlorophyll. The precipitate was filtered off and the filtrate was further concentrated. The mother liquor was concentrated and applied to a Sephadex LH-20 column. Three fractions of I (ca. 500 g), II (203 g), and III (639 g) were obtained by eluting with water that contained increasing amounts of methanol. MCI-gel CHP-20P was used to afford geraniin (23 g) and elaeocarpusin (18.8 g) from a part (50 g) of fraction III, mainly composed of geraniin and elaeocarpusin. The structure of EL was confirmed based on spectroscopic and physical data (**Figure 1A** and Supplementary Figure 1). The purity of the isolated ellagitannins was more than 98%.

### Preparation of RPMCs

Peritoneal mast cells were isolated from SD rats (Kim H.H. et al., 2014). In short, two rats were anesthetized with CO_2_ and thereafter, the peritoneal cavity was filled with 50 mL Tyrode’s buffer A using the syringe. After injection, the peritoneal cavity was massaged for approximately 2 min, opened carefully using scissors. Peritoneal cells were aspirated using a Pasteur pipette. Cells were centrifuged at 150 *g* at room temperature for 10 min, then collected and resuspended in Tyrode’s buffer A. This suspension was filtered through a centrifuge at 400 *g* for 15 min at room temperature into a Histodenz solution to separate mast cells from the other major peritoneal cells (macrophages and small lymphocytes). The supernatant containing other cells were discarded, and mast cells in the pellet were washed and resuspended. The purity of mast cells was approximately 95% based on toluidine blue staining. More than 97% of the cells were viable based on trypan blue staining (Supplementary Figure 2).

### Preparation of Mouse BMMCs

Bone marrow-derived mast cells (BMMCs) were isolated from male ICR mouse as previously described (Rodriguez Cetina Biefer et al., 2018). After removing the skin from the hip to ankle, muscles were carefully removed from the femur of mouse without breaking the bones with scissors. Thereafter, the long bones of the second leg were prepared in the same manner. All subsequent steps were performed into the sterile cell culture hood to prevent contamination of the bone marrow. All equipment was prepared under clean, sterile conditions. In the next step, the femur and tibia were rinsed with PBS. Finally, the end of each bone was cut off to open the bone marrow cavity, and the bone marrow was flushed with 1 mL medium using an insulin syringe to collect cells in a Petri dish (Sarstedt, Nümbrecht, Germany). The cell suspension was transferred into a T flask.

### Histamine Assay

Blood samples were centrifuged at 400 *g* for 15 min at 4°C, and then serum was collected. Anti-DNP IgE (50 ng/mL) sensitized RBL-2H3 cells and BMMCs (5 × 10^5^ cells/well in 12-well plates) were incubated overnight. Cells were washed three times in PBS, before treating with EL. After 1 h, DNP-HSA (100 ng/mL) was treated to stimulate mast cells for either 4 h or 30 min. HMC-1 cells (1 × 10^6^ cells/well in 24-well plates) were pretreated with or without EL for 30 min and then stimulated with PMA (20 nM) and calcium inophore A23187 (PMACI) for 8 h. Isolated RPMCs were incubated in 24-well plates (2 × 10^4^ cells) in the presence of anti-DNP IgE (50 ng/mL). For a complete description of the methods used in the histamine experiments, please see the Supplementary Materials.

### Passive Cutaneous Anaphylaxis (PCA)

IgE-mediated PCA model was established (Kim et al., 2017). Anti-DNP IgE (0.5 μg) was injected into the ears of the mice (*n* = 5/group, total *n* = 25) and incubated for 48 h. EL was dissolved in an acetone/olive oil (1:3) solution and was painted at doses of 10 and 100 ng/ear, 1 h before antigen challenge. Each mouse received antigen challenge by intravenously injecting 1 μg DNP-HSA containing 4% Evans blue (1:1) into the tail vein, and euthanized after 30 min. The ears were dissected and transferred into 1 mL of 1 M KOH. The reaction of KOH was stopped using the 4 mL of a 5:13 mixture of acetone and phosphoric acid after 1 day. The amount of dye was measured using spectrophotometer at 620 nm.

### Active Systemic Anaphylaxis (ASA)

Mice (*n* = 5/group, total *n* = 25) were sensitized with the OVA mixture [100 μg of OVA and 2 mg of alum adjuvant in 200 μL of phosphate-buffered saline (PBS)] by intraperitoneal injection on day 0 and day 7 as previously described (Kim et al., 2017). Subsequently, EL (1, 10 mg/kg) and Dexa (10 mg/kg) were orally administered on days 9, 11, and 13. On day 14, 200 μg of OVA was intraperitoneally injected and rectal temperature was measured every 10 min for 90 min. After 90 min, a blood sample was obtained from the abdominal artery of each mouse.

### Statistical Analysis

Statistical analyses were performed using GraphPad Prism 5 (GraphPad Software, Inc., San Diego, CA, United States). Treatment effects were analyzed using ANOVA followed by Duncan’s multiple range tests. A value of *p* < 0.05 was considered statistically significant.

## Results

### Effects of EL on Degranulation in Mast Cells

The chemical structure of EL is shown in **Figure 1A**. The cytotoxicity of EL was determined by colorimetric analysis based on the MTT assay. Various concentrations (0.01–50 μM) of EL were treated with RBL-2H3 and HMC-1 cells for 12 h. At concentrations less than 10 μM EL, cell viability was not affected (**Figure 1B**). We evaluated the effect of EL on the degranulation of mast cells based on measurements of histamine and β-hexosaminidase release. RBL-2H3 cells and BMMCs activated with DNP-HSA released large amounts of granule-related mediators (histamine and β-hexosaminidase), and this release was reduced by EL treatment in a concentration-dependent manner (**Figures 1C–F**). The primary cultured RPMCs and HMC-1 also showed an inhibitory effect on histamine release by EL (**Figures 1G,H**). Dexa was used as a positive control drug.

**FIGURE 2:**
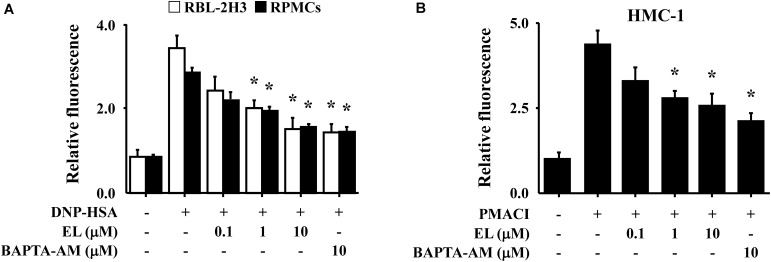
Effects of EL on intracellular calcium. **(A)** After overnight anti-DNP IgE incubation, RBL-2H3 cells (5 × 10^5^/well) and RPMCs (2 × 10^4^/well) were incubated with Fluo-3/AM for 1 h, treated with or without EL for 1 h, and then challenged with DNP-HSA. **(B)** HMC-1 cells (1 × 10^6^/well) were pre-incubated with EL for 30 min prior to incubation with PMACI for 5 min. Intracellular calcium was detected using a fluorescent plate reader. Graph data represent the mean ± SEM of three independent experiments. ^∗^*p* < 0.05. Dexa, dexamethasone.

Calcium is a prime messenger in mast cell activation signaling (Izquierdo et al., 2014). To investigate the mechanism by which EL causes a reduction in mast cell degranulation, we analyzed intracellular calcium. In particular, the fluorescence indicator Fluo-3/AM was used to investigate inhibition of calcium influx by EL. The incubation of RBL-2H3 cells or RPMCs with EL (0.1–10 μM) decreased intracellular calcium levels in a concentration-dependent manner (**Figure 2A**). These effects were confirmed in HMC-1 cells. PMACI-stimulated HMC-1 cells had a significantly elevated intracellular calcium concentration and a reduced calcium concentration in the EL-treated group (**Figure 2B**).

### Effects of EL on Inflammatory Cytokine Expression and NF-κB Activation in Mast Cells

**FIGURE 3:**
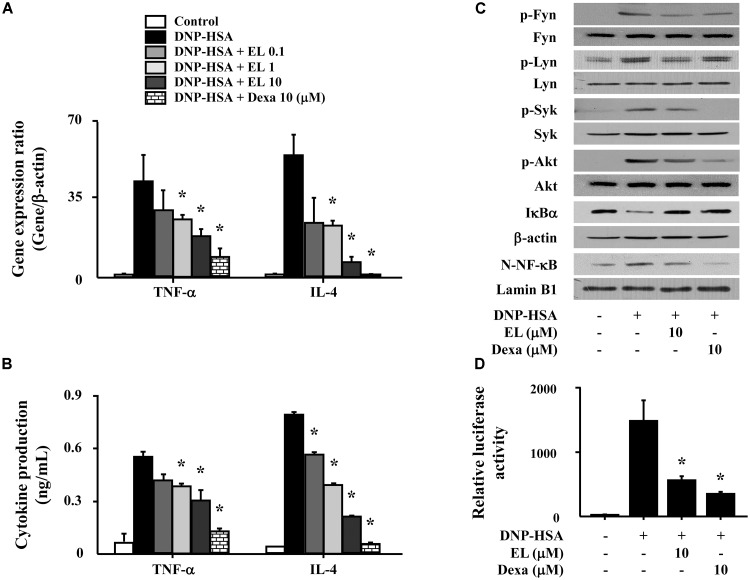
Effects of EL on the expression and secretion of inflammatory cytokines and the activation of signaling proteins and NF-κB. RBL-2H3 cells (5 × 10^5^/well) were sensitized with anti-DNP IgE (50 ng/mL). After overnight incubation, the cells were pretreated with or without drugs, including EL and Dexa, for 1 h, and then challenged with DNP-HSA (100 ng/mL). **(A)** The gene expression of inflammatory cytokines was determined by qPCR. **(B)** The secretion of inflammatory cytokines was measured by ELISA. Graph data represent the mean ± SEM of three independent experiments. RBL-2H3 cells (1.5 × 10^6^/well) were sensitized with anti-DNP IgE (50 ng/mL). After overnight incubation, the cells were pretreated with or without EL for 1 h and then challenged with DNP-HSA (100 ng/mL). **(C)** The activation of signaling proteins and NF-κB was assayed by western blot (N, nuclear). b-Actin and lamin B were used as loading controls. The band is a representative of three independent experiments. **(D)** NF-κB-dependent transcriptional activity was determined by luciferase activity assay. ^∗^*p* < 0.05 compared with the DNP-HSA-challenged group. Dexa, dexamethasone.

We used RBL-2H3 cells to confirm the effects of EL on the expression of pro-inflammatory cytokines (TNF-α and IL-4). Cells stimulated with DNP-HSA produced high levels of all cytokines after 1 h. EL (0.1–10 μM) suppressed the gene expression and secretion of cytokines in a concentration-dependent manner (**Figures 3A,B**). To elicit an inhibitory mechanism of pro-inflammatory cytokines, activation of NF-κB was assessed. In our results, nuclear translocation of NF-κB and degradation of IκBα in DNP-HSA stimulated RBL-2H3 cells were observed significantly suppressed by EL. We investigated the effects of EL on the activation of typical Fc𝜀RI signaling proteins (Fyn, Lyn, Syk, and Akt) in mast cells in order to predict the target pathways that the effects of suppression of the expression of pro-inflammatory cytokines by EL. When DNP-HSA-challenged RBL-2H3 cells were pretreated with EL, the activation of Fyn, Lyn, Syk, and Akt was inhibited (**Figure 3C**). In addition, the suppressive effect of EL on NF-κB activation was confirmed using a reporter assay. EL suppressed NF-κB-luciferase activity in mast cells (**Figure 3D**).

### Effects of EL on Local and Systemic Anaphylaxis

**FIGURE 4:**
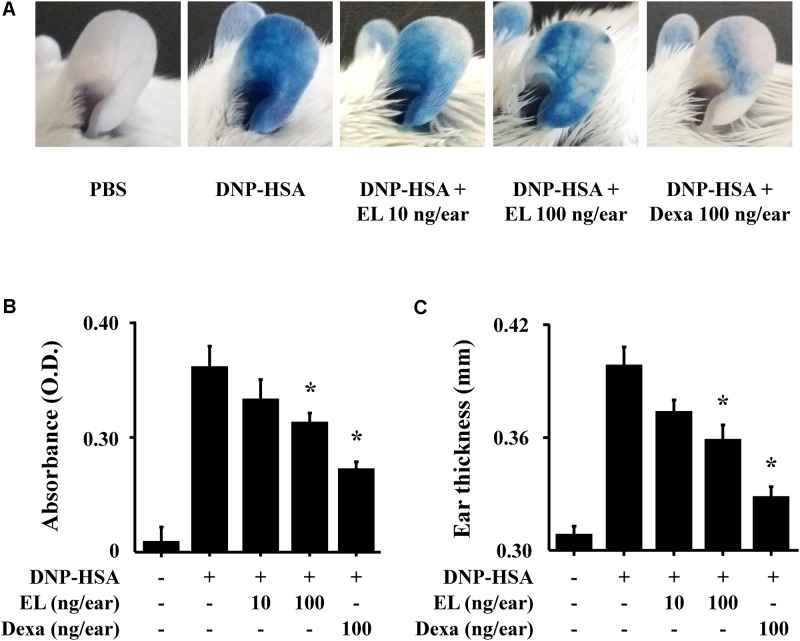
Effects of EL on IgE-mediated passive cutaneous anaphylaxis. Mouse ear skin (*n* = 5/group) was sensitized with an intradermal injection of anti-DNP IgE (0.5 μg/site) for 48 h. EL and Dexa were applied 1 h before the intravenous injection of DNP-HSA (1 mg/mouse) and 4% Evans blue (1:1) mixture. **(A)** Representative photographic images of ears. **(B)** Each amount of the dye was extracted as described in Section “Materials and Methods” and detected suing a spectrophotometer. **(C)** Ear thickness was measured with a dial thickness gauge. Graph data represent the mean ± SEM (*n* = 5/group) of two independent experiments. ^∗^*p* < 0.05 compared with the DNP-HSA-challenged group. Dexa, dexamethasone.

To examine the effects of EL on IgE-mediated allergic reaction *in vivo*, a PCA model was used. The PCA model is mainly used as an *in vivo* model of local allergic reaction (Bae et al., 2011). Before injection, EL (10 or 100 ng/ear) or Dexa (100 ng/ear) was painted on the ears of mice. When injected with 4% Evans blue dye mixed with antigen, Evans blue was poured out from the PCA reaction site. This confirms that the vascular permeability is vastly increased. EL significantly attenuated the PCA reaction based on Evans blue extravasation (**Figures 4A,B**). The increase in ear thickness induced by antigen injection was also reduced by EL treatment (**Figure 4C**).

**FIGURE 5:**
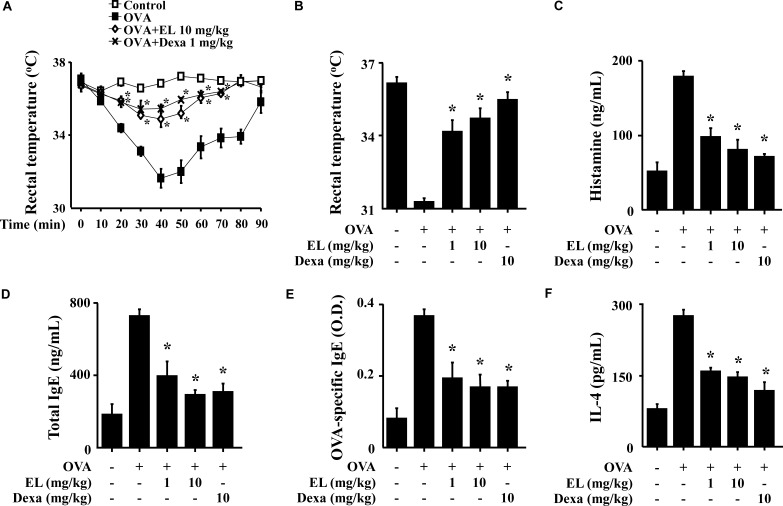
Effects of EL on ovalbumin-induced active systemic anaphylaxis (ASA). The induction of systemic anaphylaxis and oral administration of drugs, including EL and Dexa, are described in Section “Materials and Methods.” Blood was obtained from the abdominal artery of each mouse and measurement of serum histamine, total IgE, OVA-specific IgE, and IL-4 were conducted. **(A)** Rectal temperature was measured every 10 min for 90 min. **(B)** Rectal temperature of the mice at 40 min. **(C–F)** Serum levels of histamine, total IgE, OVA-specific IgE, and IL-4. Graph data represent the mean ± SD (*n* = 5/group) of two independent experiments. ^∗^*p* < 0.05 compared with the OVA-challenged group. Dexa, dexamethasone.

The systemic anaphylaxis model is widely used to examine immediate-type hypersensitivity, which is strongly associated with mast cells (Marone et al., 2002). Mice were sensitized through repetitive administrations of OVA with alum adjuvant, and anaphylaxis was induced by challenge with OVA. After an intraperitoneal injection of OVA, mice were monitored for 90 min. Over a period of 20–40 min, body temperature was decreased, while the serum histamine level was considerably increased. The observed decrease in rectal temperature was reduced by oral administration of EL, and the serum histamine level was also diminished (**Figures 5A–C**). Total serum IgE, OVA-specific IgE, and IL-4 levels were increased after the challenge with OVA and decreased by EL (**Figures 5D–F**).

## Discussion

In the present study, we investigated the inhibitory effects of EL, an ellagitannin isolated from *E. sylvestris*, on mast cell-mediated allergic inflammation. Previous structural studies on hydrolysable tannins showed that ellagitannins are biosynthetically derived from gallotannins (Takashi et al., 1986). In our previous research, gallotannin isolated from *Euphorbia* species inhibited the activation of NF-κB as well as mast cell-mediated allergic inflammation (Kim et al., 2009; Park et al., 2010). EL has been characterized as a brownish ellagitannin upon nitrous acid treatment. By the ^1^H and ^13^C NMR spectra, EL contains 4,4′, 5,5′, 6,6′-hexahydroxydiphenoyl and galloyl ester groups and a carbohydrate moiety. In the ^1^H NMR spectrum, the sugar 1-H was abnormally deshielded by esterification, the chemical shift (δ 6.51) almost coincided with the chemical shift of geraniin (δ 6.56). The observed pattern of the smaller coupling constants (≈4 Hz) for geraniin and sugar protons were also similar (Takashi et al., 1986) (Supplementary Figure 1).

IgE-mediated allergic reactions through the Fc𝜀RI receptor is known to be the main mechanism of mast cell activation (Krystel-Whittemore et al., 2015). In this study, we investigated whether EL inhibits mast cell degranulation using murine and human cell lines (RBL-2H3 and HMC-1) and primary cultured mast cells (BMMCs and RPMCs). Primary cells retain many functions *in vivo*, and also endogenously express the target of the drug (Fang, 2014). BMMCs and RPMCs are the most widely used primary mast cells (Solis-Lopez et al., 2017). BMMCs cultured for 4 weeks in the presence of recombinant murine IL-3 produce virtually pure (>95%) Fc𝜀RI^+^, c-kit^+^ mast cells that can keep growing for at least 10 weeks. RPMCs are mature serosal-type mast cells and their responses to IgE differ quantitatively and qualitatively from BMMC responses (Malbec et al., 2007). HMC-1 lack Fc𝜀RI on the cell surface, unlike other cells. Therefore, PMACI was used to stimulate HMC-1. Despite these differences, the overall release of histamine by mast cells was reduced by EL.

The most relevant source of histamine in the immune system is mast cells. Histamine, which is stored in cytoplasmic granules together with serotonin, proteases, proteoglycans, cytokines/chemokines, and angiogenic factors, which are rapidly released by various stimuli (Borriello et al., 2017). Previous study showed that the inhibition of histamine release is associated with intracellular calcium levels (Weng et al., 2012). Thus, we focused on calcium signaling in mast cells. In our study, EL showed the ability to reduce levels of intracellular calcium. The intracellular calcium responses induced by Fc𝜀RI cross-linking are important for mast cell degranulation (Tshori and Razin, 2010). Therefore, EL showed inhibition of mast cell degranulation through calcium blockade and the low level of histamine that results from blocked calcium diminishes the allergic reaction.

Calcium is also defined as an important secondary messenger to induce the expression of pro-inflammatory cytokines in mast cells (Jeong et al., 2002; Olenchock et al., 2006; Kang et al., 2018). Pro-inflammatory cytokines, including TNF-α and IL-4, are known to stimulate various cells to produce inflammatory mediators (McLeod et al., 2015; Gao et al., 2017). This study found that EL inhibited IgE-induced TNF-α and IL-4 expression in mast cells. Pro-inflammatory cytokines are stored in secretory granules within mast cells. After cross-linking Fc𝜀RI of IgE and antigen, TNF-α and IL-4 are rapidly released during stimulation (Stanley and Lacy, 2010). IL-4 induces class switching in B-cells to induce IgE production and also activates T-cell development. TNF-α can induce physiological immune reactions by triggering leukocyte infiltration and tissue fibrosis and promoting pro-inflammatory states through the secretion of cytokines (Kim M. et al., 2014). Thus, EL suppresses the expression of TNF-α and IL-4, suggesting that EL can modulate mast cell-mediated allergic inflammatory responses.

The production of pro-inflammatory cytokines is primarily regulated by NF-κB, a main transcription factor involved in the allergic inflammatory response. Many studies have shown that NF-κB is an important factor in the regulation of various immune responses in allergic diseases, such as allergy and atopic dermatitis (Choi et al., 2013; Kim et al., 2017). NF-κB has long been considered a prototypical pro-inflammatory signaling pathway (Lawrence, 2009). Moreover, mast cells are activated through the phosphorylation of Lyn or the Fyn/Syk pathway and activated mast cells induce the release of a variety of inflammatory mediators (TNF-α, leukotrienes, and prostaglandins) (Lee et al., 2013; Do et al., 2017). Syk is present in the endoplasmic reticulum (ER), is mainly in the tyrosine-phosphorylated form because of autophosphorylation, and can induce downstream signaling. Phosphorylation of Syk leads to its activation, and activates the membrane adaptor LAT1 and LAT2 (NTAL) proteins. As a scaffold for multimolecular signaling complexes, phosphorylated LAT directly or indirectly binds with cytosolic adaptors, such as Gads, Grb2, SLP-76, SHC and the enzymes PLCγ1 and PLCγ2 (Siraganian et al., 2010). PLCγ is the key signaling molecule that utilizes the conversion of phosphatidylinositol bisphosphate (PIP_2_) to second messengers, inositol triphosphate (IP_3_) and diacylglycerol (Alsaleh et al., 2016). Binding of IP_3_ to its receptor induces the influx of extracellular calcium (Ma and Beaven, 2009). In support of this, EL significantly reduced the phosphorylation of Lyn and Fyn, which are downstream proteins in the Fc𝜀RI receptor cascade (Roth et al., 2008). These findings suggest that EL acts as an inhibitor for the signaling transduction pathways involved in Fc𝜀RI crosslinking-mediated mast cell activation.

As stated earlier, mast cells are the major effector cells associated with various allergic responses that can release inflammatory mediators, such as histamine and pro-inflammatory cytokines (Galli, 2000). Previous studies have shown that histamine, cytokines, and proteases released by mast cells play an important role in allergic inflammation in mast cell-deficient mice (Lee, 2016). An animal model of passive cutaneous allergic reactions characterized by ear swelling has been well-accepted for the evaluation of the allergic response (Joo et al., 2015). PCA is commonly used immediate allergic response model, which is characterized by increased vascular permeability. Evans blue, a dye that binds with extravasated plasma albumin, is injected to visualize and quantify the increased permeability characteristic of PCA (Pettersson et al., 2017). Thus, EL-mediated reduction in pigmentation and ear swelling that are observed in the PCA model is suggested to arise from the suppression of allergic responses. In addition, sensitization with OVA enhances IgE production in the serum, after which re-exposure to OVA initiates an allergic response through the binding of antigen and IgE-receptor complexes on the surface of mast cells (Lim and Kim, 2014; Chen et al., 2016). In particular, hypothermia, an allergic response to OVA challenges, is caused by increased serum histamine levels (Kim et al., 2017). Therefore, the OVA-induced ASA model is an appropriate animal model for mast cell-mediated type I hypersensitivity (Finkelman et al., 2005). In our experiments, the ASA was inhibited by the EL treatment. From these results, we suggest that EL suppresses the allergic reaction by inhibiting mast cell activation.

Although there have been reports about the pharmacological effects of *E. sylvestris*, its anti-allergic effects have not been elucidated. Our results demonstrated the mechanisms responsible for the anti-allergic inflammatory activity of EL. We provided evidence for the contribution of EL to the prevention or treatment of mast cell-mediated allergic inflammatory disorders. It should be noted that there are many drug candidates for the treatments of allergic diseases including various chemical drugs, herbs, and herbal extracts. In our results, the anti-allergic inflammatory effects of EL was comparable with dexamethasone, the positive control drug at the same dose throughout *in vitro* and *in vivo*. In conclusion, we propose that EL could be a candidate for the treatment of allergic inflammatory responses by reducing the activation of mast cells; nevertheless, further investigations would be necessary to elucidate the direct binding target of EL.

## Ethics Statement

The care and treatment of the animals were in accordance with the guidelines established by the Public Health Service Policy on the Humane Care and Use of Laboratory Animals and were approved by the Institutional Animal Care and Use Committee of Kyungpook National University (IRB #2016-0050).

## Author Contributions

M-JK carried out major experiments and drafted the manuscript. Y-AC and Y-YK carried out the qPCR and Western blot. M-CB and BL provided input for the statistical planning and evaluation of data. P-HP, T-YS, and TK supported with the study design, reviewed the protocol, and participated in interpretation of the primary outcome. DK and S-HK provided critical input to the manuscript. All authors commented on the study and approved the final version of the manuscript.

## Conflict of Interest Statement

The authors declare that the research was conducted in the absence of any commercial or financial relationships that could be construed as a potential conflict of interest.

## Abbreviations

NF: nuclear factor
PCA: passive cutaneous anaphylaxis
TNF: tumor necrosis factor
