# Supplementary material for: Elaeocarpusin Inhibits Mast Cell-Mediated Allergic Inflammation
Source: Front Pharmacol. 2018 Jun 7;9:591. doi: 10.3389/fphar.2018.00591 (PMC5999758; doi:10.3389/fphar.2018.00591)
Supplement: Supplementary file 1 [file Image_1.PDF]

Elaeocarpusin Inhibits Mast Cell-Mediated Allergic Inflammation

Methods

Animals

Male Imprinting Control Region (ICR) mice (6-weeks-old) and male Sprague–Dawley (SD) rats (10-weeks-old) were purchased from the Dae-Han Experimental Animal Center (Daejeon, Korea). All animals had *ad libitum* access to standard rodent chow and filtered water. Throughout the study, animals were housed in a laminar air flow room in a controlled environment (temperature, 22°C ± 2°C; relative humidity, 55% ± 5%; 12 h/12-h light/dark cycle). The care and treatment of the animals were in accordance with the guidelines established by the Public Health Service Policy on the Humane Care and Use of Laboratory Animals and were approved by the Institutional Animal Care and Use Committee of Kyungpook National University (IRB #2016-0050).

Reagents and cell culture

Anti-dinitrophenyl (DNP) IgE, DNP-human serum albumin (HSA), ovalbumin (OVA), dexamethasone (Dexa), Histodenz, phorbol 12-myristate 13-acetate (PMA), calcium ionophore A23187, and *o*-phthaldialdehyde were purchased from Sigma (St. Louis, MO). Alum adjuvant was procured from Thermo Scientific (Waltham, MA). RBL-2H3, HMC-1, rat peritoneal mast cells (RPMCs), and bone marrow-derived mast cells (BMMCs) were grown at 37°C in an atmosphere of 5% CO_2 in Iscove's modified Dulbecco's medium, DMEM, α-MEM (Gibco, Grand Island, NY) and RPMI 1640 (Hyclone, Logan, UT), respectively, supplemented with 100 units/mL penicillin–streptomycin and 10% heat-inactivated FBS (Gibco). In addition,

complete RPMI 1640 media was supplemented by 4 mM L-glutamine, 25 M HEPES, 50 μM 2-mercaptoethanol, 1 mM sodium pyruvate, MEM nonessential amino acid solution (Gibco), 10 ng/mL murine IL-3, and 2 ng/mL murine stem cell factor (PeproTech EC, London, UK).

Cell viability

Cell viability was determined by colorimetric analysis using 3-(4,5-dimethylthiazol-2-yl)-2, 5-diphenyltetrazolium bromide (MTT) (Je et al., 2015). RBL-2H3 cells (6×10^4 cells/well in a 96-well plate) were treated with EL for 12 h, followed by incubation with MTT reagent for 2 h. The formed formazan crystals were dissolved in DMSO and the absorbance was read at 570 nm using a spectrophotometer (Molecular Devices, Sunnyvale, CA).

β-hexosaminidase

RBL-2H3 cells and BMMCs (5×10^5 cells/well in 12-well plates) sensitized with anti-DNP IgE (50 ng/mL) were washed three times in PBS, treated with EL for 1 h, and then stimulated with DNP-HSA (100 ng/mL) for either 1 h or 30 min. After incubation, cells were centrifuged at 150 *g* for 5 min at 4°C and separated from the medium. Then, 40 μL supernatant was transferred to a 96-well plate and incubated at 37°C for 1 h with 40 μL 0.1 M citrate buffer (pH 4.5) containing 1 mM 4-nitrophenyl-N-acetyl-β-D-glucosaminide. Cells were lysed with 0.5% Triton X-100 and the supernatant was used to measure the total β-hexosaminidase activity. The absorbance was measured with a spectrophotometer (Molecular Devices) at 405 nm.

Histamine assay

After 24 h, cells were washed with PBS, treated with EL for 1 h, and then stimulated with DNP-HSA (100 ng/mL) for 30 min. Cells were centrifuged at 150 *g* for 5 min at 4°C and separated from the medium. To measure histamine in the supernatant, 0.1 N HCl and 60%

perchloric acid were added to the sample and centrifuged. The supernatant was transferred to a 1.5 mL Eppendorf tube, 5 M NaCl, 5 N NaOH, and *n*-butanol were added, and the solution was vortexed and centrifuged again. The supernatant was mixed with 0.1 N HCl and *n*-heptane and then centrifuged. Histamine in the aqueous layer was measured as previously described using *o*-phthaldialdehyde spectrofluorometry (Je et al., 2015). Fluorescence intensity was detected using a fluorescence plate reader (Molecular Devices) at an excitation wavelength of 380 nm and an emission wavelength of 440 nm.

Intracellular calcium

Intracellular calcium was measured using the fluorescent indicator Fluo-3/AM (Invitrogen, Carlsbad, CA) (Kim et al., 2014). RBL-2H3 cells (6×10^4 cells/well in 96-well plates) were sensitized by overnight exposure to anti-DNP IgE (50 ng/mL). HMC-1 cells (1×10^5 cells/well in 96-well plates) and RPMCs (1×10^4 cells/well in 96-well plates) were seeded. The cells were then incubated with Fluo-3/AM for 1 h at 37°C and washed with Tyrode's buffer (137 mM NaCl, 5.5 mM glucose, 12 mM $NaHCO_3$, 2.7 mM KCl, 0.2 mM NaH_2PO_4, 1 mM $MgCl_2$, and 1.8 mM $CaCl_2$) to remove the dye from the cell surface. RBL-2H3 cells were then pretreated with or without EL for 1 h prior to antigen challenge with DNP-HSA (100 ng/mL). As a positive control, BAPTA-AM (Calbiochem, La Jolla, CA) was used as a calcium chelator. Fluorescent intensity was detected using a fluorescent plate reader at an excitation wavelength of 485 nm and an emission wavelength of 510 nm. The intracellular calcium level of untreated control cells was assigned a relative absorbance value of 1. Morphological evaluation of cells was performed using an Olympus IX71 inverted microscope (Olympus America, Melville, NY). Digital images were acquired using a Magnafire SP (Olympus) digital camera.

Enzyme-linked immunosorbent assay (ELISA)

Cytokine levels were measured by ELISA. RBL-2H3 cells (5×10^5 cells/well in a 12-well plate) were sensitized with overnight exposure to anti-DNP IgE (50 ng/mL). The cells were pretreated with EL for 1 h prior to a challenge with DNP-HSA (100 ng/mL) for 6 h. The ELISA was performed on a 96-well Nunc Immuno Plate using specific kits (BD Biosciences, San Diego, CA) according to the manufacturer's protocol. After the substrate reaction was terminated, the absorbance was measured using a spectrophotometer at a wavelength of 450 nm.

qPCR

After stimulation with DNA-HSA with or without EL, RBL-2H3 cells were seeded at a density of 5×10^5 cells/well in a 12-well plate. Total cellular RNA was isolated using a RNAiso Plus kit (Takarabio, Shiga, Japan) according to the manufacturer's protocol. The first-strand complementary DNA (cDNA) was synthesized using a PCR kit (Thermo Scientific). The primer sets were selected using the Primer3 program (Whitehead Institute, Cambridge, MA). The number of cycles was optimized so that product accumulation was within the exponential range. qPCR was performed according to the manufacturer's protocol using a Thermal Cycler Dice TP850 (Takarabio). Briefly, 2 μL cDNA (100 ng), 1 μL sense and antisense primer solution (0.4 μM), 12.5 μL SYBR Premix Ex Taq (Takarabio), and 9.5 μL of nuclease free water were mixed together to obtain a final volume of 25 μL in each reaction tube. Relative quantification of mRNA expression was performed using TP850 software. The primer sequences used were as follows: TNF-α (F: 5´-TCC CAA ATG GGC TCC CTC TC-3´, R: 5´-AAA TGG CAA ACC GGC TGA CG-3´), IL-4 (F: 5´-TGC ACC GAG ATG TTT GTA CCA GA-3´, R: 5´-TTG CGA AGC ACC CTG GAA G-3´), and β-actin (F: 5´-GAA GCT GTG CTA TGT TGC CCT AGA-3´, R: 5´-GTA CTC CTG CTT GCT GAT CCA CAT-3´). The conditions used for PCR were as previously described (Kim et al., 2017).

Western blotting

Nuclear and cytosolic proteins were extracted as previously described (Choi et al., 2013). Anti-DNP IgE (50 ng/mL)-sensitized RBL-2H3 cells (1.5×10^6 cells/well in a 6-well plate) were washed three times with PBS, treated with EL for 1 h, and then stimulated with DNP-HSA (100 ng/mL) for 7 min, (Fyn, Lyn and Syk), 30 min (Akt), or 1 h (IκBα and NF-κB). Cells were washed with PBS, resuspended in 100 μL cell lysis buffer A (0.5% Triton X-100, 150 mM NaCl, 10 mM HEPES, 1 mM EDTA/Na_3VO_4, 0.5 mM PMSF/DTT, and 5 μg/mL leupepin/aprotinin), vortexed, incubated on ice for 5 min, and centrifuged at 400 *g* at 4°C for 5 min. The supernatant was collected and used as the cytosolic protein extract. The pellets were washed three times with 1 mL PBS, suspended in 25 μL cell lysis buffer B (25 % glycerol, 420 mM NaCl, 20 mM HEPES, 1.2 mM $MgCl_2$, 0.2 mM EDTA, 1 mM Na_3VO_4, 0.5 mM PMSF/DTT, and 5 μg/mL leupeptin/aprotinin), vortexed, sonicated for 30 s, incubated on ice for 20 min, and centrifuged at 15,000 *g* for 15 min at 4°C. The supernatant was collected and used as the nuclear protein extract. The same amounts of cell protein were electrophoresed using a 7.5–10% SDS-polyacrylamide gel and transferred to a nitrocellulose membrane. After blocking, the membrane was incubated with a primary antibody for the target and then with anti-IgG horseradish peroxidase-conjugated secondary antibody. The following antibodies were purchased from Santa Cruz Biotechnology (Santa Cruz, CA): NF-κB (sc-109, rabbit polyclonal, 1:1000), IκBα (sc-371, rabbit polyclonal, 1:1000), β-actin (sc-8432, mouse monoclonal, 1:1000), and lamin B (sc-6217, goat polyclonal, 1:1000). The following antibodies were purchased from Abcam (Cambridge, UK); phospho-Fyn (ab182661, rabbit monoclonal, 1:500), Fyn (ab125016, rabbit monoclonal, 1:1000). The following antibodies were purchased from Cell Signaling Technology (Beverly, MA); phospho-Lyn (#2731, Tyr507,

rabbit polyclonal, 1:1000), phospho-Syk (#2711, Tyr525/526, rabbit polyclonal, 1:1000), phospho-Akt (#9271, Ser473, rabbit polyclonal, 1:1000), Lyn (#2732, rabbit polyclonal, 1:1000), Syk (#2712, rabbit polyclonal, 1:1000), Akt (#9272, rabbit polyclonal, 1:1000). Immunodetection was performed using a chemiluminescent substrate (Thermo Scientific).

Transient transfection and luciferase activity assay

For transient transfections, RBL-2H3 were seeded at 5×10^5 in a 12-well plate one day before transient transfection. The expression vectors containing the NF-κB luciferase reporter construct (pNF-κB-LUC, plasmid containing NF-κB binding site; Stratagene, Grand Island, NY) were transfected with serum- and antibiotics-free opti-minimum essential medium containing 2 μL Lipofectamine 2000 reagent (Invitrogen). After 5 h of incubation, the medium was replaced with DMEM containing 10% FBS and antibiotics. Cells were allowed to recover at 37°C for 12 h and subsequently were stimulated as indicated. Cell lysates were prepared and analyzed for luciferase activity using the Luciferase Assay System (Promega, Madison, WI), according to the manufacturer’s instructions.

Supplementary Figure 1. ^{13}C NMR characteristics of elaeocarpusin

Group	Carbon	δ
Glucose	C-1	92.4
	C-2	68.8
	C-3	74.4
	C-4	63.5
	C-5	74.4
	C-6	64.4
Galloyl	C-1	120.3
	C-2	110.5
	C-3	146.1
	C-4	139.7
	C-5	146.1
	C-6	110.5

Group	Carbon	δ
Hexahydroxydiphenoyl	C-1,1′	116.5
		116.7
	C-2,2′	123.1
		125.0
	C-3,3′	109.3
		110.1
	C-4,4′	144.7
		144.9
	C-5,5′	137.4
		137.5
	C-6,6′	145.1
		145.4

Group	Carbon	δ	Carbon	δ	Carbon	δ
2,4-Acyl group	C-1	51.8	C-1′	116.1	C-1″	170.7
	C-2	49.8	C-2′	118.5	C-2″	80.7
	C-3	38.0	C-3′	114.1	C-3″	109.1
	C-4	197.7	C-4′	147.6	C-4″	89.4
	C-5	96.3	C-5′	136.2	C-5″	73.7
	C-6	108.1	C-6′	148.6	C-6″	76.5

Supplementary Figure 2. Representative photomicrographs of mast cells stained with toluidine blue and trypan blue.

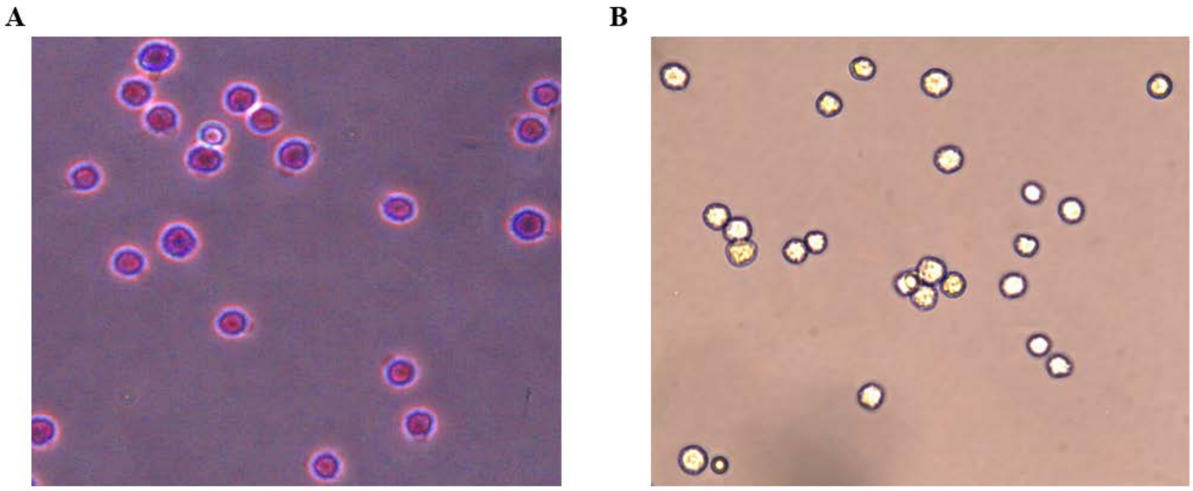

Isolated RPMCs were diluted in PBS (1:10). After dilution, cells were stained in a ratio 1:1 with dye. Representative photomicrographs of RPMCs stained with (A) toluidine blue and (B) trypan blue (magnification of ×400). Data represent the mean value at 10 random sites of each stain.

References

Choi, J. K., Oh, H. M., Lee, S., Park, J. W., Khang, D., Lee, S. W., et al. (2013). Oleanolic acid acetate inhibits atopic dermatitis and allergic contact dermatitis in a murine model. Toxicol Appl Pharmacol. 269, 72-80. doi: 10.1016/j.taap.2013.03.001

Je, I. G., Kim, H. H., Park, P. H., Kwon, T. K., Seo, S. Y., Shin, T. Y., et al. (2015). SG-HQ2 inhibits mast cell-mediated allergic inflammation through suppression of histamine release and pro-inflammatory cytokines. Exp Biol Med (Maywood). 240, 631-638. doi: 10.1177/1535370214555663

Kim, H. H., Park, S. B., Lee, S., Kwon, T. K., Shin, T. Y., Park, P. H., et al. (2014). Inhibitory effect of putranjivain A on allergic inflammation through suppression of mast cell activation. Toxicol Appl Pharmacol. 274, 455-461. doi: 10.1016/j.taap.2013.12.006

Kim, Y. Y., Je, I. G., Kim, M. J., Kang, B. C., Choi, Y. A., Baek, M. C., et al. (2017). 2-Hydroxy-3-methoxybenzoic acid attenuates mast cell-mediated allergic reaction in mice via modulation of the FcεRI signaling pathway. Acta Pharmacol Sin. 38, 90-99. doi: 10.1038/aps.2016.112
